# Effects of short-term ambient PM_2.5_ exposure on cardiovascular disease incidence and mortality among U.S. hemodialysis patients: a retrospective cohort study

**DOI:** 10.1186/s12940-022-00836-0

**Published:** 2022-03-11

**Authors:** Yuzhi Xi, David B. Richardson, Abhijit V. Kshirsagar, Timothy J. Wade, Jennifer E. Flythe, Eric A. Whitsel, Geoffrey C. Peterson, Lauren H. Wyatt, Ana G. Rappold

**Affiliations:** 1 Department of Epidemiology, Gillings School of Global Public Health, University of North Carolina at Chapel Hill, Chapel Hill, NC USA; 2 Center for Public Health and Environmental Assessment, Oak Ridge Institute for Science and Education at the United States Environmental Protection Agency, Chapel Hill, NC USA; 3 Division of Nephrology and Hypertension, Department of Medicine, UNC School of Medicine, University of North Carolina Kidney Center, Chapel Hill, NC USA; 4 Center for Public Health and Environmental Assessment, Office of Research and Development, U.S. Environmental Protection Agency, Chapel Hill, NC USA; 5 Department of Medicine, School of Medicine, University of North Carolina at Chapel Hill, Chapel Hill, NC USA; 6 Center for Public Health and Environmental Assessment Public Health, Chemical and Pollution Assessment Division, United States Environmental Protection Agency, Washington, DC, USA

**Keywords:** Hemodialysis patients, Susceptible population, Air pollution, PM_2.5_, Short-term exposure

## Abstract

**Background:**

Ambient PM_2.5_ is a ubiquitous air pollutant with demonstrated adverse health impacts in population. Hemodialysis patients are a highly vulnerable population and may be particularly susceptible to the effects of PM_2.5_ exposure. This study examines associations between short-term PM_2.5_ exposure and cardiovascular disease (CVD) and mortality among patients receiving maintenance in-center hemodialysis.

**Methods:**

Using the United State Renal Data System (USRDS) registry, we enumerated a cohort of all US adult kidney failure patients who initiated in-center hemodialysis between 1/1/2011 and 12/31/2016. Daily ambient PM_2.5_ exposure estimates were assigned to cohort members based on the ZIP code of the dialysis clinic. CVD incidence and mortality were ascertained through 2016 based on USRDS records. Discrete time hazards regression was used to estimate the association between lagged PM_2.5_ exposure and CVD incidence, CVD-specific mortality, and all-cause mortality 1 t adjusting for temperature, humidity, day of the week, season, age at baseline, race, employment status, and geographic region. Effect measure modification was assessed for age, sex, race, and comorbidities.

**Results:**

Among 314,079 hemodialysis patients, a 10 µg/m^3^ increase in the average lag 0–1 daily PM_2.5_ exposure was associated with CVD incidence (HR: 1.03 (95% CI: 1.02, 1.04)), CVD mortality (1.05 (95% CI: 1.03, 1.08)), and all-cause mortality (1.04 (95% CI: 1.03, 1.06)). The association was larger for people who initiated dialysis at an older age, while minimal evidence of effect modification was observed across levels of sex, race, or baseline comorbidities.

**Conclusions:**

Short-term ambient PM_2.5_ exposure was positively associated with incident CVD events and mortality among patients receiving in-center hemodialysis. Older patients appeared to be more susceptible to PM_2.5_-associated CVD events than younger hemodialysis patients.

**Supplementary Information:**

The online version contains supplementary material available at 10.1186/s12940-022-00836-0.

## Background

Short-term exposure to PM_2.5_ (particles with a mass median aerodynamic diameter of less than 2.5 µm) is an established cause of cardiovascular disease and mortality [1], and is a criteria air pollutant regulated by US National Ambient Air Quality Standards (NAAQS). When inhaled, PM_2.5_ particles can travel deep into the lung and even enter the circulatory system [2]. PM_2.5_ particles can trigger inflammation (both systemic and local) and oxidative stress, accelerate atherosclerosis, and alter cardiac autonomic function which can eventually lead to cardiovascular diseases and ultimately deaths [3–8].

Previous studies have established ambient PM_2.5_ as a cause of cardiovascular disease and mortality in the general population; however, less is known about the risks associated with PM_2.5_ exposure among subgroups of the population that may be particularly susceptible to PM_2.5_ -associated health effects [1, 9]. One such group are patients with kidney failure requiring dialysis. These individuals have a higher cardiovascular disease (CVD) risk than the population, and US dialysis patients have an annual all-cause mortality risk of 16% [9–13]. Quantifying the mortality risk from air pollution is a critical step in identifying modifiable adverse health outcome risk factors in this population. Yet, to our knowledge, there has been no prior investigation of the short-term effects of ambient PM_2.5_ on CVD incidence or mortality in this patient population.

Therefore, we assessed the association of short-term ambient PM_2.5_ exposure with new CVD events, and with mortality among patients receiving maintenance in-center hemodialysis (HD) in the contiguous United States, considering lagged effects of exposure (up to 3 days after exposure) and potential effect measure modifications by age, sex, race, and baseline comorbidities.

## Methods

### Study setting and study population

We identified a retrospective cohort of maintenance hemodialysis patients from the United States Renal Data System (USRDS), a claim-based national registry of renal disease patients. USRDS includes nearly all dialysis patients in the United States with individual level data on patients’ demographics, hospitalizations, and provider information [14]. Baseline demographic characteristics, dialysis treatment, and comorbidity status were extracted. We included patients aged 18 years or older who initiated in-center maintenance hemodialysis treatment between Jan 1^st^ 2011 and Dec 31^st^ 2016 in the contiguous U.S. who had Medicare as primary payer. Participants were followed from the fourth month of dialysis initiation until first incident diagnosis of CVD, change of renal disease treatment modality (e.g. receiving kidney transplant), loss of Medicare coverage, death, or administrative end of study follow-up (Dec 31^st^ 2016).

### Exposure assessment and weather data

For each participant-day under follow-up, a daily record of location, at the ZIP code-level, was assigned based on the ZIP code of the dialysis clinic visited during that period. Patients typically visited dialysis clinics every 2 to 3 days. For a date between claim reporting periods (with no information on clinic visits), the ZIP code of the last clinic visited was assigned (1.3% of the person-days in the analysis had a ZIP code that was different from the previous person-day of the same individual). Previous studies have shown that patients in the USRDS cohort have a median travel distance of 5.7 miles to their dialysis clinic [15, 16]. Thus, ZIP code assignment based on dialysis clinic location should be a relatively accurate surrogate for hemodialysis patients’ geographic location of residence during study follow-up.

ZIP code-level daily PM_2.5_ concentrations were estimated with a previously described prediction model [17, 18]. In brief, this model estimates daily average PM_2.5_ on a 1×1 km grid for the entire contiguous U.S. by incorporating multiple inputs like chemical transport model simulations, satellite aerosol optical depth measurements, meteorology, and land use. Gridded PM_2.5_ estimates were then converted to population-weighted ZIP-level estimates using 2010 Census tract population values. Daily temperature and relative humidity data were estimated based on records from the U.S. National Oceanic and Atmospheric Administration (NOAA). In detail, daily temperature and relative humidity from weather station measurements were interpolated to census tract centroids and then averaged over tracts to ZIP codes. A patient’s daily record of location was linked to an estimate of the daily concentration of PM_2.5_ at that location, as well as an estimate of the temperature and relative humidity for that date and ZIP code.

### Outcome assessment

Follow-up to ascertain incident CVD events, CVD-related deaths, and vital status through December 31, 2016 was based on USRDS records [19]. The outcomes of interest were all-cause mortality, CVD-specific mortality, and incident CVD event. The primary cause of death recorded in ESRD Death Notification Form (CMS 2746) was used to classify mortality into all-cause and CVD-specific mortality. We defined incident CVD events as the first CVD related emergency department (ED) visit, hospitalization, or mortality, which ever occurred first since the follow-up start. It is worth noting that, conventionally, “incident CVD event” or “CVD incidence” refers to those first events occurring among participants determined to be free of CVD at baseline only, and in this analysis, these terms’ meaning is different from their conventional definition by expanding to all participants regardless of their baseline CVD status. International Classification of Disease, 9^th^ and 10^th^ Revision (ICD-9, ICD-10) codes were used to identify cause-specific ED visit and inpatients cardiovascular events. In brief, any ED and hospitalization with a code indicating cerebrovascular, ischemic heart disease, hypertensive disease, heart failure, cardiac arrest, and related incidence were classified as CVD related ED and hospitalization events. The cause of death codes and International Classification of Disease codes used are summarized in Supplemental Table 1.

### Statistical analysis

We utilized discrete time hazards models to assess the association between ZIP code-level short-term PM_2.5_ exposure with mortality and CVD events [20]. Discrete time hazards models have been widely used in occupational and environmental cohort studies [21–23]. This approach is similar to a Poisson regression model applied to a person-period data structure, which allows controlling for individual level factors [20]. The equation for the fully-adjusted model is presented in Eq. 1, where Y is the individual specific response at time t (e.g. 0 if no event, 1 event….) and *i, t, and k* index individual, time (day), and number of lag days respectively. We used a generalized linear model E(Y_it_| covariates) = g^{-1}(${\eta }_{it}$) with a complementary log–log link function g and a linear predictor ${\eta }_{it}$ as follows:

1 $$\normalsize log (-\mathrm{log}(1-\mathrm{E}({\mathrm{Y}}_{it}|\mathrm{ Covariates}))={\eta }_{it}= {factor(MonthSinceDialysis)_i} + {{\sum }_{k=0}^{K}\beta }_{1k}{PM2.5}_{itk}+{\beta }_{2}{Temperature}_{it}+{\beta }_{3}{RelativeHumidity}_{it}+{\beta }_{4}{factor(DOW)}_{it}+{\beta }_{5}{factor(Season)}_{it}+{\beta }_{6}{Age(Dialysis Initiation)}_{i}+{\beta }_{7}{factor(Race)}_{i}+{\beta }_{8}{factor(EmploymentStatus)}_{i}+{\beta }_{9}{factor(Region)}_{i}$$

Variables DOW, Season, Race, Region and Employment status were categorical thus the coefficients represent vectors rather than scalars. Hazard ratios (HRs) per 10-µg/m^3^ increase in PM_2.5_, together with corresponding 95% confidence intervals (95% CIs) were estimated. We incorporated time since initiation of dialysis in the model as the analysis time scale to control for the potential temporal variation in baseline risk of the outcome of interest by this factor, which has been suggested to be an important predictor of mortality among dialysis patients in prior studies [24, 25]. Time-varying confounders including: daily temperature, daily relative humidity, day of the week (DOW), and season (warm vs. cold) were controlled for to adjust for potential time varying confounding. Specifically, DOW and season were incorporated to account for short- and long-term temporal variability of the outcomes [26, 27]. We tested non-linear functional forms of temperature and relative humidity in the model and found no significant change in effect estimates and model fitness when compared to linear forms. Thus, we modeled temperature and relative humidity as linear variables in the final model. Time-invariant confounders included age at dialysis initiation, race, baseline employment status, and geographic region (Northeast, Midwest, South, and West). Baseline employment status (employed, unemployed, retired, other) was incorporated as a surrogate for socio-economic status (SES).

Short-term PM_2.5_ exposure effects were assessed for the same day, lag days, and moving averages across different lag periods to assess the persistent effect of PM2.5 exposure across multiple days. The same day (lag day 0) effects were assessed in a model for that same day PM_2.5_ and its association with the outcome. The lagged effects of prior days’ exposures were assessed in a model that associated lag day 0, 1, 2, and 3 exposures with the outcome in the same model. The average lagged effects of prior exposure days were assessed in a model that associated the average of daily PM_2.5_ across lag periods 0–1, 0–2, or 0–3 days prior. For example, the moving average of lag period 0–1 was calculated by averaging the exposure levels of the day of the event and the day preceding the event, which is later incorporated in the model as the sole exposure variable. Among different models assessing short-term lagged effect, the model with the lower Akaike information criterion (AIC) value was considered to be the better model depicting the short-term effects.

Potential effect measure modification by baseline comorbidity status, race, sex, and age at dialysis initiation were assessed. The comorbidities assessed were diabetes, CVD, and chronic obstructive pulmonary disease (COPD) reported at the start of maintenance hemodialysis on CMS 2728 form. Specifically, the patients who reported to have any of the following conditions: atherosclerotic heart disease (ASHD), cardiac arrest, congestive heart failure (CHF), cerebrovascular disease, cardiac dysrhythmia, ischemic heart disease (IHD), myocardial infarction (MI), pericarditis, peripheral vascular disease, and other cardiac disease, were classified to have CVD at baseline. The patients who reported to have diabetes (primary or contributing), who were diabetic and currently on insulin, on oral medications, or without medications, or with diabetic retinopathy were classified to have diabetes at baseline. The patients who reported to have chronic obstructive pulmonary disease were classified to have COPD at baseline.

Likelihood ratio tests for homogeneity were performed by comparing models with and without an interaction term between the potential modifier and PM term. A small Chi-square test of likelihood ratios test p-value (less than 0.05) suggested the rejection of effect homogeneity hypothesis, which indicates PM effects are not similar across different levels of potential modifiers.

The data management and statistical analysis were performed with SAS software, Version 9.4 (SAS Institute Inc., Cary, NC, USA). Plots were generated with R Project for Statistical Computing Version 3.5.0 (R Foundation for Statistical Computing, Vianna, Austria). The study protocol was reviewed and exempted by the Institutional Review Board of the University of North Carolina on Nov 16, 2020 (Study #:20–1469).

## Results

The final cohort included a total of 314,079 hemodialysis patients with 193,121,928 person-days of follow-up in the mortality analysis and 94,385,773 person-days in the CVD event analysis. Between 2011 and 2016, there were 82,567 deaths, 35,857 (43.4%) of which were CVD-related mortalities, and 208,113 patients experienced at least one CVD event.

Table 1 displays the study population characteristics. At the initiation of hemodialysis, more than half of the participants were older than 65 years of age. The average vintage period, which is the time between dialysis initiation and the end of follow-up, was 1.9 (SD = 1.4) years. Approximately 44% of the study participants were female, and 65% were white. At hemodialysis initiation, more than half of the cohort was reported to have diabetes, about 60% was reported to have CVD, and 10% was reported to have COPD. Patients who died during the study period were likely to be older at dialysis initiation compared to the overall study population or the cohort who experienced at least one CVD event.

**Table 1:** Study population baseline characteristics overall and by outcomes of interests

**Characteristics**	Total	Mortality		Incidence
		All-Cause	CVD-Specific	Incident CVD Event
**n**	314,079	82,567	35,857	208,113
**Person-day**	193,121,928	193,121,928	193,121,928	94,385,773
**Age at HD Initiation (year)**	63.6 (14.6)	69.0 (13.0)	69.4 (13.2)	65.4 (14.0)
**Age at HD Initiation**				
* 18–44 years*	34,191 (10.9)	3,803 (4.6)	1,808 (5.0)	17,019 (8.2)
* 45–64 years*	119,140 (37.9)	23,565 (28.5)	10,759 (30.0)	74,068 (35.6)
* 65–74 years*	81,741 (26.0)	23,978 (29.0)	10,280 (28.7)	57,125 (27.5)
* 75 years and older*	79,007 (25.2)	31,221 (37.8)	13,010 (36.3)	59,901 (28.8)
**Vintage (year)**^**a**^	1.9 (1.4)	1.7 (1.2)	1.7 (1.2)	2.2 (1.5)
**Vintage (year)**				
* Less than 1 year*	108,957 (34.7)	32,153 (38.9)	13,596 (37.9)	54,144 (26.0)
* 1 – 2 years*	81,617 (26.0)	23,347 (28.3)	10,145 (28.3)	55,742 (26.8)
* 2 – 3 years*	53,198 (16.9)	14,035 (17.0)	6,266 (17.5)	40,457 (19.4)
* 3 – 4 years*	35,163 (11.2)	8,058 (9.8)	3,644 (10.2)	28,315 (13.6)
* Over 4 years*	35,144 (11.2)	4,974 (6.0)	2,206 (6.2)	29,455 (14.2)
**BMI (kg/m**^**2**^**)**	29.9 (8.1)	29.0 (8.0)	29.3 (8.0)	29.8 (8.2)
**Female**	131,209 (43.9)	36,343 (44.0)	15,244 (42.5)	93,832 (45.1)
**Race**				
* White*	194,790 (65.1)	58,106 (70.4)	25,103 (70.0)	136,196 (65.4)
* Black*	88,415 (29.6)	20,071 (24.3)	8,917 (24.9)	60,123 (28.9)
* Other*	15,868 (5.3)	4,390 (5.3)	1,837 (5.1)	11,794 (5.7)
**Reported Cause of Kidney Failure**				
* Diabetes*	152,106 (48.4)	40,205 (48.7)	18,734 (52.3)	105,544 (50.7)
* Glomerulonephritis*	20,846 (6.6)	3,536 (4.3)	1,368 (3.8)	11,351 (5.5)
* Hypertension*	97,204 (31.0)	26,555 (32.2)	11,530 (32.2)	64,554 (31.0)
* Other*	43,923 (14.0)	12,271 (14.9)	4,225 (11.8)	26,664 (12.8)
**Access Type**				
* AVF*	54,008 (18.1)	11,094 (13.4)	4,937 (13.8)	32,914 (15.8)
* Graft*	9,527 (3.2)	2,427 (2.9)	1,070 (3.0)	6,543 (3.1)
* Catheter*	235,042 (78.6)	66,320 (80.3)	28,782 (80.3)	159,809 (76.8)
* Other*	496 (0.2)	139 (0.2)	61 (0.2)	327 (0.2)
**Baseline Comorbidities**				
* CVD*	160,760 (53.8)	53,452 (64.7)	24,218 (67.5)	123,121 (59.2)
* COPD*	30,417 (10.2)	11,841 (14.3)	5,220 (14.6)	24,067 (11.6)
* Diabetes Mellitus*	178,489 (59.7)	49,725 (60.2)	22,728 (63.4)	128,114 (61.6)
**Geographic Region**				
* Northeast*	68,668 (21.9)	20,183 (24.4)	8,831 (24.6)	47,707 (22.9)
* Midwest*	59,243 (19.9)	15,362 (18.6)	6,033 (16.8)	39,405 (18.9)
* West*	40,001 (12.7)	9,091 (11.0)	4,146 (11.6)	24,398 (11.7)
* South*	146,167 (46.5)	37,931 (45.9)	16,847 (47.0)	96,603 (46.4)
**Baseline Employment Status**				
* Employed*	30,746 (9.8)	4,621 (5.6)	2,082 (5.8)	15,655 (7.5)
* Retired*	196,525 (62.6)	61,159 (74.1)	26,353 (73.5)	139,909 (67.2)
* Unemployed*	76,338 (24.3)	15,088 (18.3)	6,793 (10.9)	47,033 (22.6)
* Other*	10,470 (3.3)	1,699 (2.1)	629 (1.8)	5,516 (2.7)

^a^Vintage is defined as the time between hemodialysis initiation and the end of study follow-up, which is death, drop-out of Medicare, change of modality, or the last day of study period

*HD* Hemodialysis, *BMI* Body Mass Index, *AVF* Arteriovenous Fistula, *COPD* Chronic obstructive pulmonary disease

The distribution of ZIP code-level PM_2.5_ concentration is summarized in Supplemental Table 2. For the mortality analysis, the average daily ZIP code-level PM_2.5_ was around 8.5 (± 4.8) µg/m^3^ with an inter-quartile range (IQR) of 5.2 µg/m^3^. For CVD incidence analysis, the PM_2.5_ exposure distribution was similar to that of the mortality analysis with a mean of 8.6 (± 4.9) µg/m^3^ and IQR of 5.3 µg/m^3^.

**Fig. 1:**
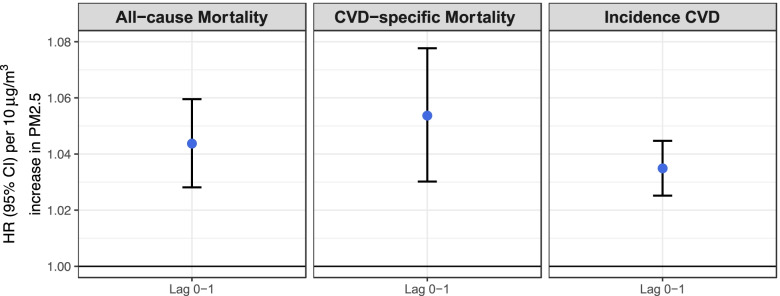
Hazard Ratios (95% CI) of short-term ambient PM_2.5_ exposure (average daily PM_2.5_ Lag 0–1) on all-cause mortality, CVD-specific mortality, and CVD incidence among HD patients, 2011–2016. The reported effect estimates were obtained from models that adjusted for time since dialysis initiation, temperature, relative humidity, day of the week (DOW), season (warm vs cold), age at dialysis initiation, race, employment status at dialysis initiation, and geographic region (Northeast, Midwest, South, and West) at dialysis initiation

Exposure to short-term daily ambient PM_2.5_ was associated with an increased hazard of all-cause mortality (Fig. 1, Supplemental Table 3). Both immediate (same day) and lagged short-term PM_2.5_ effects were observed. The HR associated with a 10-µg/m^3^ increase in same day, lag-day 1, lag-day 2, and lag-day 3 PM_2.5_ were 1.01 (95% CI: 0.99, 1.03), 1.03 (95% CI: 1.01, 1.06), 1.00 (95% CI: 0.97, 1.02), and 1.00 (95% CI: 0.98, 1.02), respectively (Fig. 1, Supplemental Table 3). The short-term PM effects were most prominent for the same day and lag-day 1 exposure. The HR associated with a 10-µg/m^3^ of the average daily PM_2.5_ across same-day and lag-day 1 was 1.04 (95% CI: 1.03, 1.06) (Fig, 1). The HRs associated with average daily PM_2.5_ across same-day and lag-day 2, up to lag-day 3 were similar to the HR of average daily PM_2.5_ across same-day and lag-day 1 (Supplemental Table 3).

**Fig. 2:**
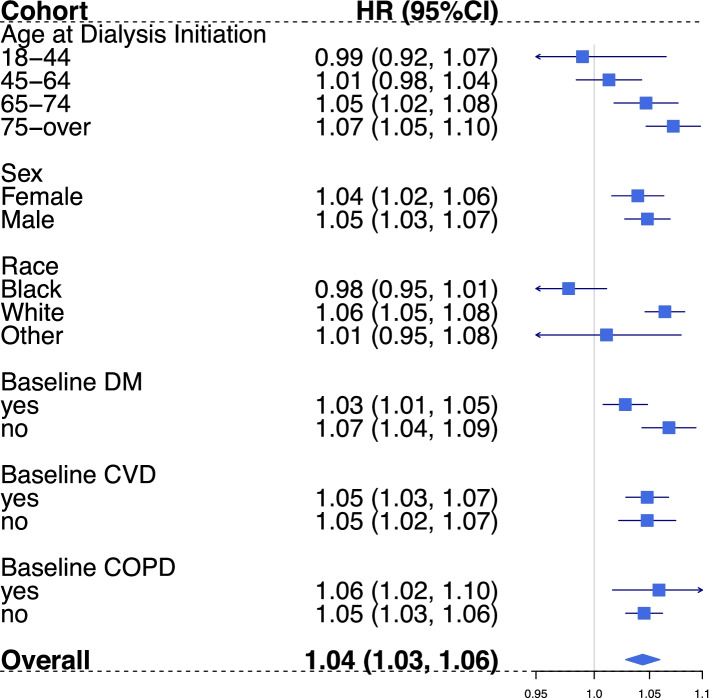
All-cause mortality stratification analysis by age at dialysis initiation, sex, race, vascular access type, and comorbidity status at dialysis initiation. Estimates (HRs per 10 µg/m^3^ increase) of association between average daily PM_2.5_ (Lag 0–1) exposure and all-cause mortality among HD patients, 2011–2016.(30). HR = Hazard Ratio, DM = Diabetes Mellitus, COPD = Chronic obstructive pulmonary disease. Effect estimates reported for age at dialysis initiation groups were adjusted for: temperature, relative humidity, DOW, season, race, employment status, and geographic region. Effect estimates reported for race groups were adjusted for: temperature, relative humidity, DOW, season, age at dialysis initiation, employment status, and geographic region. The rest estimates were obtained from models that adjusted for: temperature, relative humidity, DOW, season, age at dialysis initiation, race, employment status, and geographic region

Patients who started dialysis in the oldest age group (≥ 75 years), were of white race, and did not have diabetes at dialysis initiation experienced relatively higher PM effects on all-cause mortality compared to other groups by potential modifiers (Fig. 2). The highest HR of 1.07 (95% CI: 1.05, 1.10) associated with every10-µg/m^3^ increase in average daily PM_2.5_ across lag-day 0 and 1 was observed among patients who initiated dialysis at the age of 75-year or older (Fig. 2). The likelihood ratio test for homogeneity also suggested PM mortality effects are not homogeneous across levels of dialysis initiating age, race, and baseline diabetes status with low p-values (Supplemental Table 4).

Exposure to short-term daily ambient PM_2.5_ was associated with an elevated hazard of CVD-specific mortality (Fig. 1, Supplemental Table 3). The HR associated with a 10-µg/m^3^ increase in same day, lag-day 1, lag-day 2, and lag-day 3 PM_2.5_ were 1.02 (95% CI: 0.99, 1.05), 1.03 (95% CI, 0.99: 1.07), 0.99 (95% CI: 0.96, 1.03), and 1.01 (95% CI: 0.98, 1.04), respectively (Supplemental Table 3). The short-term PM effects were most prominent for the same day and lag-day 1 exposure for CVD-specific mortality. The HR associated with a 10-µg/m^3^ of the average daily PM_2.5_ across same-day and lag-day 1 was 1.05 (95% CI: 1.03, 1.08) (Fig. 1). The HRs associated with average daily PM_2.5_ across same-day up to lag-day 3 which were similar to the HR of average daily PM_2.5_ across same-day and lag-day 1 (Supplemental Table 3).

**Fig. 3:**
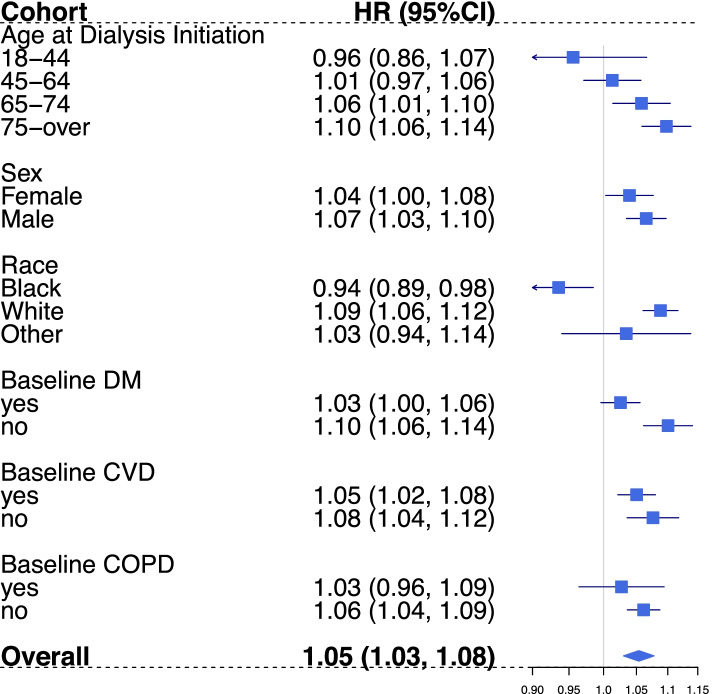
CVD-specific mortality stratification analysis by age at dialysis initiation, sex, race, and comorbidity status at dialysis initiation. Estimates (HRs per 10 µg/m^3^ increase) of association between average daily PM_2.5_ (Lag 0–1) exposure and CVD-specific mortality among HD patients, 2011–2016. HR = Hazard Ratio, DM = Diabetes Mellitus, COPD = Chronic obstructive pulmonary disease. Effect estimates reported for age at dialysis initiation groups were adjusted for: temperature, relative humidity, DOW, season, race, employment status, and geographic region. Effect estimates reported for race groups were adjusted for: temperature, relative humidity, DOW, season, age at dialysis initiation, employment status, and geographic region. The rest estimates were obtained from models that adjusted for: temperature, relative humidity, DOW, season, age at dialysis initiation, race, employment status, and geographic region

Similar to the effect measure modification effect observed for all-cause mortality, HD patients who initiated dialysis in the oldest age group (≥ 75 years), were of white race, and did not have diabetes at dialysis initiation experienced relatively higher PM effects on CVD-cause mortality compared to other groups by these potential modifiers (Fig. 3). The highest HR of 1.10 (95% CI: 1.06, 1.14) associated with 10-µg/m^3^ increase in average daily PM_2.5_ across lag day 0 and lag day 1 was observed among hemodialysis patients who initiated dialysis at the age of 75-year or older (Fig. 2). The likelihood ratio test for homogeneity also suggested the PM effect differs across levels of dialysis initiating age, race, and baseline diabetes status for CVD-specific mortality (Supplemental Table 4).

Exposure to short-term daily ambient PM_2.5_ was associated with a higher hazard of incident CVD events (Fig. 1, Supplemental Table 3). The HR associated with a 10-µg/m^3^ increase in same day, lag-day 1, lag-day 2, and lag-day 3 PM_2.5_ were 1.02 (95% CI: 1.01, 1.03), 1.01 (95% CI: 1.00, 1.03), 1.00 (95% CI: 0.98, 1.01), and 1.00 (95% CI: 0.99, 1.01), respectively (Supplemental Table 3). The short-term PM effects were most prominent for the same day and lag-day 1 exposure for CVD related events. Effects at lag-day 2 and lag-day 3 daily PM_2.5_ were not observed (Supplemental Table 3). The HR associated with a 10-µg/m^3^ of the average daily PM_2.5_ across same-day and lag-day 1 was 1.03 (95% CI: 1.03, 1.04) (Fig. 1, Supplemental Table 3). The HRs associated with long lag periods (lag 0–2 or lag 0–3) average daily PM_2.5_ was very similar to the HR of the lag 0–1 average PM_2.5_ (Supplemental Table 3).

**Fig. 4:**
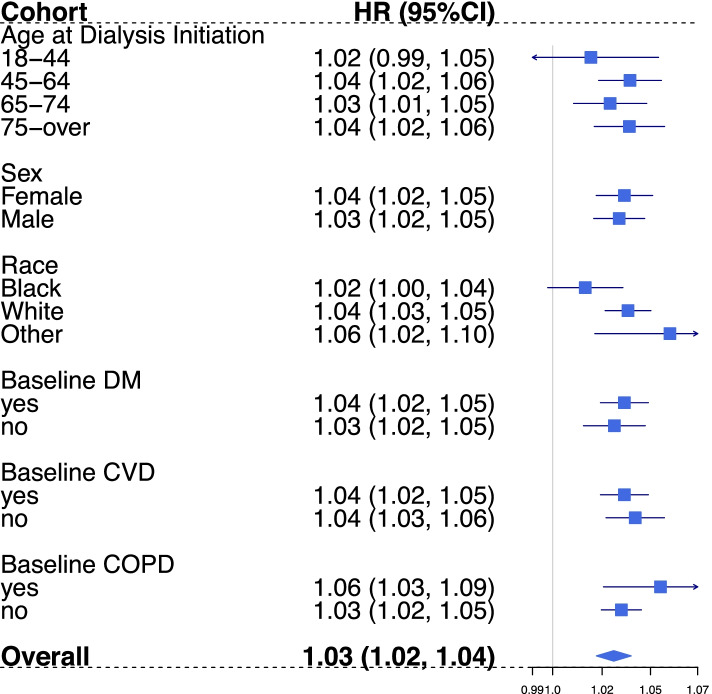
Incidence CVD events stratification analysis by age at dialysis initiation, sex, race, vascular access type, and comorbidity status at dialysis initiation. Estimates (HRs per 10 µg/m^3^ increase) of association between average daily PM_2.5_ (Lag 0–1) exposure and first CVD event since dialysis initiation among HD patients, 2011–2016. HR = Hazard Ratio, DM = Diabetes Mellitus, COPD = Chronic obstructive pulmonary disease. Effect estimates reported for age at dialysis initiation groups were adjusted for: temperature, relative humidity, DOW, season, race, employment status, and geographic region. Effect estimates reported for race groups were adjusted for: temperature, relative humidity, DOW, season, age at dialysis initiation, employment status, and geographic region. The rest estimates were obtained from models that adjusted for: temperature, relative humidity, DOW, season, age at dialysis initiation, race, employment status, and geographic region

For CVD events, none of the factors assessed significantly modified the short-term PM effect. Slightly higher hazards were observed among hemodialysis patients who had COPD at baseline and who were males with overlapping confidence interval ranges (Fig. 4). The likelihood ratio test of homogeneity also indicates the PM_2.5_ effects were similar across different levels of dialysis initiating age, race, and baseline diabetes status for incident CVD events with greater than 0.05 p-values (Supplemental Table 4).

## Discussion

We estimated associations between short-term PM_2.5_, all-cause mortality, CVD-specific mortality, and incident CVD among US hemodialysis patients during the period 2011–2016. Short-term PM_2.5_ exposure was associated with elevated hazards for all-cause mortality, CVD-specific mortality, and incident CVD among the study population. Each 10 µg/m^3^ increase in short-term PM_2.5_ was associated with a 4%, 5%, and 3% increase in the hazard of all-cause mortality, CVD-specific mortality, and incident CVD events, respectively. We also observed relatively higher PM_2.5_ hazards on all-cause mortality and CVD-specific mortality among hemodialysis patients who initiated dialysis at an older age, who were white, and who did not report having diabetes mellitus at the initiation of dialysis.

The effects of PM_2.5_ exposure among US hemodialysis patients have not been extensively evaluated. To our knowledge, this is the first study to examine short-term ambient PM_2.5_ exposure effects among US hemodialysis patients. We previously observed an elevated mortality risk associated with wildfire generated PM_2.5_ exposure among maintenance hemodialysis patients [28]. In addition to mortality, we also observed an elevated CVD incidence hazard associated with short-term PM_2.5_ exposure, which is consistent with a study among hemodialysis patients on the association between hospital admission and county-level short-term PM_2.5_ for cardiovascular causes for the same-day PM_2.5_ exposure [29]. Wyatt et al. reported a 0.9% (95% CI, 0.4, 1.8%) increase in hospital admission risk for cardiovascular causes for the same-day PM_2.5_ exposure [29]. Recently, a study demonstrated an elevated risk of all-cause mortality associated with long-term (annual average) PM_2.5_ among older (over 65-year old) US hemodialysis and peritoneal dialysis patients [30]. Their study reported that for very 10 µg/m^3^ increase in annual average PM_2.5_ exposure below 12 µg/m^3^, there is a 1–5% increase in the HR of all-cause mortality, a similar range to our findings [30]. However, it was unexpected that the association between PM_2.5_ and incident CVD was slightly lower than between PM_2.5_ and CVD-specific mortality (3% vs. 5% increase in HR per 10 µg/m3 increase in PM_2.5_, albeit with overlapping confidence intervals) because it is logical that a person would develop one or more CVD incidence(s) before he or she can die from it. However, this could be due to the different diagnosis coding regimes used to define CVD-specific mortality vs. CVD incidence. CVD mortalities were defined using CMS 2746 codes, which are not ICD-9 or -10 codes used for CVD incidence, and future studies should identify and use additional data sources on cause of death. Another possible explanation is that PM_2.5_ exposure could impact the CVD case fatality rate, which could be assessed in future studies instead of first CVD event since follow-up to take all incident CVD events into account and to further understand PM’s impact on cardiovascular health among this population. In addition, evidence is emerging that elevated levels of fine particulate matter may contribute to incident chronic kidney disease, and its progression to severe kidney function impairment that requires maintenance dialysis treatment [31–33]. Together with previously published studies, our findings support the conclusion that there is an elevated health hazard associated with particulate matter exposure among dialysis patients. Furthermore, the daily ZIP code-level PM_2.5_ for the majority (Q3 of 10.6 µg/m^3^) of the included person-days in this analysis were below the current NAAQS regulatory standard of 35 µg/m^3^ (24-h, 98^th^ percentile). On average, PM_2.5_ has been decreasing nationally in the US over the last decade [34]. However, the PM concentration in many highly-populated urban areas still exceed the NAAQS, and extreme events like wildfires also lead to short-term high-level exposures. Our findings of elevated hazards of mortality and CVD conditions suggest that reduction of individual-level exposure may be beneficial to at risk individuals such as dialysis patients, a group that continues to increase in size in the US.

Our findings suggest that there is larger magnitude of association between PM_2.5_ and mortality (both all-cause and CVD-specific) among patients who initiated dialysis treatment at an older age compared to younger dialysis initiators (Figs. 2 and 3). The highest PM_2.5_ effects were observed among the oldest age group (75 or older at the initiation of dialysis). This effect modification observed could be explained by the higher prevalence of pre-existing conditions and higher mortality risk among this age group [35, 36]. To our knowledge, only one previous study has assessed the effect of PM exposure on outcomes by age groups among dialysis patients. Feng et al. observed a higher long-term PM_2.5_ exposure effect on all-cause mortality among dialysis patients who aged 75 years or over at dialysis initiation compared to those who initiated dialysis 65–74 years [30]. Even though Feng et al.’s study population was different from our analysis, and they included peritoneal dialysis patients, our findings agrees that kidney disease patients who initiated dialysis at the age of 75 or older were likely to be particularly susceptible to PM_2.5_ exposure for all-cause mortality. This finding adds to the evidence that older hemodialysis patients should be targeted for exposure mitigation to reduce the overall public health burden in association with PM exposure.

In addition to older dialysis initiators, the results also suggest a larger magnitude of association between PM_2.5_ and mortality among white patients than non-white patients. This difference association by race could be partially explained by differences in age distribution of dialysis patients by race (Supplemental Table 5). About 29.5% (15.8% in black, and 23.9% in other race group) of white patients initiated hemodialysis at age 75 years or older. Thus, it is likely that the higher PM effect observed among white patients could be driven by the larger proportion of white patients that initiated dialysis at an older age. Feng et al. reported a slightly higher long-term mortality effect among white patients for the area of annual PM_2.5_ less than 12 µg/m^3^ but a lower effect among white patients when restricted the analysis to area of annual PM_2.5_ over 12 µg/m^3^ [30]. Studies in other populations have also reported mixed results by race stratification [1]. In addition, this racial mortality hazards variation could be explained by the fact that the baseline mortality risks are different across race groups for ESKD patients. In a large cohort study on US dialysis patients, Yan et al. observed a consistently higher mortality risk among non-Hispanic whites in comparison to Hispanics and non-Hispanic blacks regardless of age [35]. Thus, it is not conclusive to determine how race is a modifier for PM_2.5_ shot-term mortality effect, and more studies are needed to determine whether and how race modifies the association for dialysis patients as a social factor.

We also observed an un-expected higher hazard among patients with no diabetes at baseline. This difference in all-cause and CVD-specific mortalities hazards by baseline diabetes status could be partially explained by the fact that more patients with diabetes (50.4% vs. 46.5%) initiated dialysis at a younger age (before 65-year old) (Supplemental Table 5). To our knowledge, no study has assessed the short-term particulate matter exposure effect by baseline diabetes status among dialysis patients. Feng et al.’s study on long-term PM effect, observed a higher risk among patients who reported diabetes as primary reason of end-stage kidney disease, which is different from baseline diabetes status [30]. Furthermore, studies among general population yields mixed and non-significant results regarding the potential modification by diabetes status on PM_2.5_ effect [1, 37–39]. Thus, more studies are needed to determine comorbidity specific effect for fine particle exposure among dialysis patients.

This study has several strengths. First, the USRDS database is highly representative of hemodialysis patients in the US, with a nearly complete inclusion of those who initiated in-center hemodialysis between 2011and 2016 in the US. The USRDS registry provides a complete registry of all deaths and healthcare service usages and contains detailed information regarding demographics, comorbidities, and dialysis clinic information. Second, linkage of the exposure using the dialysis treatment date and ZIP code of dialysis clinic visited enabled the most accurate exposure classification we can achieve with the available data. In our study population, more than half of the included patients had at least one ZIP code-level change of location during follow-up. This suggests that taking location changes into account for exposure linkage is necessary to avoid exposure misclassification for this population. Third, we utilized a sophisticated model of high spatial and temporal resolution with established accuracy to estimate the ZIP code-level daily PM_2.5_ concentrations for the US [18, 40]. Last, the use of discrete time hazards model allowed for control for individual level time invariant factors together with time-varying factors to better estimate the health risk associated with fluctuation in ambient PM_2.5_. This study design also allowed us to assess effect measure modification by individual-level factors (e.g. race, age).

This study also has some limitations. First, exposure misclassification is always a concern in environmental exposure studies as the individual-level exposure is not known. Instead, we assumed that the average exposure is similar across patients within in the same ZIP code area and that the degree of exposure misclassification does not vary simultaneously with daily variation in ZIP code-level exposure and health risks. Second, in this analysis we used information on employment at dialysis initiation to classify socio-economic status, which could result in some misclassification of SES. Employment at dialysis initiation may not necessarily reflect the patients’ SES. However, this is the best information available in our source data. Future studies should identify additional source of information or collect information for individual SES classification (e.g. income, education) among dialysis patients to better understand the potential confounding or modification effect by this factor. Third, information on additional potential effect measure modifiers was not available. Effect measure modification assessment for other relevant health risk factors such as blood pressure, dialysis adequacy,, rest diuresis, liver diseases, dyslipidemia, cancer, vascular access type, and CV risk score should be performed to understand what other factors may modify the association between PM exposure and health outcomes. Finally, for all observational studies there is the possibility of unmeasured confounding (e.g. individual physical activity). In the presence of unmeasured confounding, our estimates could not be interpreted as causal.

## Conclusions

In conclusion, in this large retrospective open cohort study, we found evidence of short-term effects of ambient PM_2.5_ exposure on mortality and CVD outcomes among patients receiving maintenance in-center HD. For every 10 unit increase in ambient PM_2.5_ the mortality and CVD hazards increased by 3–5%, and the mortality hazards associated with PM_2.5_ was most prominent in older patients with a 10% increase in CVD-specific mortality hazard. These findings suggest that it is necessary for more studies to develop and implement interventions to manage particulate matter exposure in this and other particularly fragile populations.

## Supplementary Information

**Additional file 1: Supplemental Table 1. **Cause of death and ICD codes used to identify CVD incidences. **Supplemental Table 2.** United States, 2011-2016 ZIP code-level daily ambient PM_2.5_ concentration and meteorological data for all person-days included. **Supplemental Table 3**. Hazards ratios (95% confidence interval) estimates per 10 µg/m^3^ increase for the association between short-term PM_2.5_ exposures and all-cause mortality, CVD-specific mortality, and incident CVD events among the overall study population. **Supplemental Table 4. **LRT Test For Homogeneity Results. **Supplemental Table 5**. Study cohort age at dialysis initiation distribution (%) by potential modifiers.

## Data Availability

The datasets sued in this analysis are available from the corresponding author and United States Renal Data System (USRDS) upon request.

## Abbreviations

ASHD: Atherosclerotic heart disease
AVF: Arteriovenous fistula
BMI: Body mass index
CI: Confidence interval
CMS: Centers for Medicare & Medicaid Services
COPD: Chronic obstructive pulmonary disease
CVD: Cardiovascular disease
DOW: Day of the week
ED: Emergency department
EPA: Environmental Protection Agency
ESRD: End-stage renal disease
GEE: Generalized estimating equation
HD: Hemodialysis
HF: Heart failure
HR: Hazards ratio
ICD-10: International Classification of Disease 10th Revision
ICD-9: International Classification of Disease 9th Revision
IHD: Ischemic heart disease
IQR: Inter-quartile range
LRT: Likelihood ratio test
NAAQS: National Ambient Air Quality Standards
NOAA: National Oceanic and Atmospheric Administration
PM ISA: Integrated Science Assessment for Particulate Matter
PM2.5: Particulate matter with aero-diameter of 2.5 µm or smaller
SES: Socio-economic status
STD: Standard deviation
US: United States
USRDS: United States Renal Data System
WHO: World Health Organization

## References

[1] Us EPA, Agency USEP (2019). Integrated Science Assessment (ISA) for Particulate Matter (Final Report, Dec 2019). Washingotn.

[2] Wold LE, Simkhovich BZ, Kleinman MT, Nordlie MA, Dow JS, Sioutas C (2006). In vivo and in vitro models to test the hypothesis of particle-induced effects on cardiac function and arrhythmias. Cardiovasc Toxicol.

[3] Feng S, Gao D, Liao F, Zhou F, Wang X (2016). The health effects of ambient PM2.5 and potential mechanisms. Ecotoxicology and environmental safety..

[4] Kouassi KS, Billet S, Garçon G, Verdin A, Diouf A, Cazier F (2010). Oxidative damage induced in A549 cells by physically and chemically characterized air particulate matter (PM2.5) collected in Abidjan, Côte d'Ivoire. J Appl Toxicol.

[5] Longhin E, Holme JA, Gutzkow KB, Arlt VM, Kucab JE, Camatini M (2013). Cell cycle alterations induced by urban PM2.5 in bronchial epithelial cells: characterization of the process and possible mechanisms involved. Particle and Fibre Toxicology..

[6] Wang G, Jiang R, Zhao Z, Song W (2013). Effects of ozone and fine particulate matter (PM(2.5)) on rat system inflammation and cardiac function. Toxicology letters..

[7] Xing YF, Xu YH, Shi MH, Lian YX (2016). The impact of PM2.5 on the human respiratory system. J Thorac Dis..

[8] Nelin TD, Joseph AM, Gorr MW, Wold LE (2012). Direct and indirect effects of particulate matter on the cardiovascular system. Toxicol Lett.

[9] Cozzolino M, Mangano M, Stucchi A, Ciceri P, Conte F, Galassi A (2018). Cardiovascular disease in dialysis patients Nephrology, dialysis, transplantation official publication of the European Dialysis and Transplant Association. European Renal Association.

[10] United States Renal Data System (2018). 2018 USRDS Annual Data Report: Epidemiology of kidney disease in the United States.

[11] Lovasik BP, Zhang R, Hockenberry JM, Schrager JD, Pastan SO, Mohan S (2016). Emergency Department Use and Hospital Admissions Among Patients With End-Stage Renal Disease in the United States. JAMA Intern Med.

[12] Roberts MA, Polkinghorne KR, McDonald SP, Ierino FL (2011). Secular trends in cardiovascular mortality rates of patients receiving dialysis compared with the general population. Am J Kidney Dis.

[13] Mu Y, Chin AI, Kshirsagar AV, Zhang Y, Bang H (2018). Regional and Temporal Variations in Comorbidity Among US Dialysis Patients: A Longitudinal Study of Medicare Claims Data. Inquiry : a journal of medical care organization, provision and financing.

[14] U.S. Renal Data System. (2018). 2018 Researcher's Guide to the USRDS Database..

[15] Prakash S, Coffin R, Schold J, Lewis SA, Gunzler D, Stark S (2014). Travel distance and home dialysis rates in the United States. Peritoneal dialysis international : journal of the International Society for Peritoneal Dialysis.

[16] Stephens JM, Brotherton S, Dunning SC, Emerson LC, Gilbertson DT, Harrison DJ (2013). Geographic disparities in patient travel for dialysis in the United States. The Journal of rural health : official journal of the American Rural Health Association and the National Rural Health Care Association.

[17] Di Q, Amini H, Shi L, Kloog I, Silvern R, Kelly J (2019). An ensemble-based model of PM(2.5) concentration across the contiguous United States with high spatiotemporal resolution. Environment international..

[18] Di Q, Kloog I, Koutrakis P, Lyapustin A, Wang Y, Schwartz J (2016). Assessing PM2.5 Exposures with High Spatiotemporal Resolution across the Continental United States. Environ Sci Technol..

[19] Saran R, Robinson B, Abbott KC, Agodoa LY, Albertus P, Ayanian J, Balkrishnan R, Bragg-Gresham J, Cao J, Chen JL, Cope E, Dharmarajan S, Dietrich X, Eckard A, Eggers PW, Gaber C, Gillen D, Gipson D, Gu H, Hailpern SM, Hall YN, Han Y, He K, Hebert H, Helmuth M, Herman W, Heung M, Hutton D, Jacobsen SJ, Ji N, Jin Y, Kalantar-Zadeh K, Kapke A, Katz R, Kovesdy CP, Kurtz V, Lavalee D, Li Y, Lu Y, McCullough K, Molnar MZ, Montez-Rath M, Morgenstern H, Mu Q, Mukhopadhyay P, Nallamothu B, Nguyen DV, Norris KC, O'Hare AM, Obi Y, Pearson J, Pisoni R, Plattner B, Port FK, Potukuchi P, Rao P, Ratkowiak K, Ravel V, Ray D, Rhee CM, Schaubel DE, Selewski DT, Shaw S, Shi J, Shieu M, Sim JJ, Song P, Soohoo M, Steffick D, Streja E, Tamura MK, Tentori F, Tilea A, Tong L, Turf M, Wang D, Wang M, Woodside K, Wyncott A, Xin X, Zang W, Zepel L, Zhang S, Zho H, Hirth RA, Shahinian V. US Renal Data System 2016 Annual Data Report: Epidemiology of Kidney Disease in the United States. Am J Kidney Dis. 2017;69(3 Suppl 1):A7-A8. 10.1053/j.ajkd.2016.12.004. 10.1053/j.ajkd.2016.12.004 PMC6605045 28236831

[20] Richardson DB (2010). Discrete time hazards models for occupational and environmental cohort analyses. Occup Environ Med.

[21] Chang HH, Reich BJ, Miranda ML (2013). A spatial time-to-event approach for estimating associations between air pollution and preterm birth. J R Stat Soc Ser C Appl Stat.

[22] Gaskins AJ, Fong KC, Abu Awad Y, Di Q, Mínguez-Alarcón L, Chavarro JE (2019). Time-Varying Exposure to Air Pollution and Outcomes of in Vitro Fertilization among Couples from a Fertility Clinic. Environ Health Perspect.

[23] Peters A, von Klot S, Berglind N, Hörmann A, Löwel H, Nyberg F (2006). Comparison of different methods in analyzing short-term air pollution effects in a cohort study of susceptible individuals. Epidemiol Perspect Innov.

[24] Chertow GM, Johansen KL, Lew N, Lazarus JM, Lowrie EG (2000). Vintage, nutritional status, and survival in hemodialysis patients. Kidney Int.

[25] Sumida K, Yamagata K, Iseki K, Tsubakihara Y (2016). Different impact of hemodialysis vintage on cause-specific mortality in long-term hemodialysis patients. Nephrology, dialysis, transplantation : official publication of the European Dialysis and Transplant Association - European Renal Association.

[26] Peng RD, Dominici F, Pastor-Barriuso R, Zeger SL, Samet JM (2005). Seasonal analyses of air pollution and mortality in 100 US cities. Am J Epidemiol.

[27] Zhang H, Schaubel DE, Kalbfleisch JD, Bragg-Gresham JL, Robinson BM, Pisoni RL (2012). Dialysis outcomes and analysis of practice patterns suggests the dialysis schedule affects day-of-week mortality. Kidney Int.

[28] Xi Y, Kshirsagar AV, Wade TJ, Richardson DB, Brookhart MA, Wyatt L (2020). Mortality in US Hemodialysis Patients Following Exposure to Wildfire Smoke. J Am Soc Nephrol.

[29] Wyatt LH, Xi Y, Kshirsagar A, Di Q, Ward-Caviness C, Wade TJ (2020). Association of short-term exposure to ambient PM(2.5) with hospital admissions and 30-day readmissions in end-stage renal disease patients: population-based retrospective cohort study. BMJ Open..

[30] Feng Y, Jones MR, Chu NM, Segev DL, McAdams-DeMarco M (2021). Ambient Air Pollution and Mortality among Older Patients Initiating Maintenance Dialysis. Am J Nephrol.

[31] Bowe B, Xie Y, Li T, Yan Y, Xian H, Al-Aly Z. Particulate Matter Air Pollution and the Risk of Incident CKD and Progression to ESRD. J Am Soc Nephrol. 2018;29(1):218–30. 10.1681/ASN.2017030253. 10.1681/ASN.2017030253 PMC5748906 28935655

[32] Mehta AJ, Zanobetti A, Bind MA, Kloog I, Koutrakis P, Sparrow D (2016). Long-Term Exposure to Ambient Fine Particulate Matter and Renal Function in Older Men: The Veterans Administration Normative Aging Study. Environ Health Perspect.

[33] Lue SH, Wellenius GA, Wilker EH, Mostofsky E, Mittleman MA (2013). Residential proximity to major roadways and renal function. J Epidemiol Community Health.

[34] Particulate Matter (PM2.5) Trends (2010–2019). 2020. Cited 20 May 2021. Available from: https://www.epa.gov/air-trends/particulate-matter-pm25-trends.

[35] Yan G, Norris KC, Yu AJ, Ma JZ, Greene T, Yu W (2013). The relationship of age, race, and ethnicity with survival in dialysis patients. Clinical journal of the American Society of Nephrology : CJASN.

[36] Johansen KL, Chertow GM, Jin C, Kutner NG (2007). Significance of frailty among dialysis patients. J Am Soc Nephrol.

[37] Goldberg MS, Burnett RT, Stieb DM, Brophy JM, Daskalopoulou SS, Valois MF (2013). Associations between ambient air pollution and daily mortality among elderly persons in Montreal, Quebec. The Science of the total environment.

[38] Goldberg MS, Burnett RT, Yale JF, Valois MF, Brook JR (2006). Associations between ambient air pollution and daily mortality among persons with diabetes and cardiovascular disease. Environ Res.

[39] Zanobetti A, Dominici F, Wang Y, Schwartz JD (2014). A national case-crossover analysis of the short-term effect of PM2.5 on hospitalizations and mortality in subjects with diabetes and neurological disorders. Environmental health..

[40] Di Q, Rowland S, Koutrakis P, Schwartz J (2017). A hybrid model for spatially and temporally resolved ozone exposures in the continental United States. J Air Waste Manag Assoc..

