# Supplementary material for: Effects of short-term ambient PM2.5 exposure on cardiovascular disease incidence and mortality among U.S. hemodialysis patients: a retrospective cohort study
Source: Environ Health. 2022 Mar 11;21:33. doi: 10.1186/s12940-022-00836-0 (PMC8917758; doi:10.1186/s12940-022-00836-0)
Supplement: Supplementary file 1 — Additional file 1: Supplemental Table 1. Cause of death and ICD codes used to identify CVD incidences. Supplemental Table 2. United States, 2011-2016 ZIP code-level daily ambient PM2.5 concentration and meteorological data for all person-days included. Supplemental Table 3. Hazards ratios (95% confidence interval) estimates per 10 µg/m3 increase for the association between short-term PM2.5 exposures and all-cause mortality, CVD-specific mortality, and incident CVD events among the overall study population. Supplemental Table 4. LRT Test For Homogeneity Results. Supplemental Table 5. Study cohort age at dialysis initiation distribution (%) by potential modifiers. [file 12940_2022_836_MOESM1_ESM.docx]

**SUPPLEMENTAL MATERIALS**

**Supplemental Table 1**. Cause of death and ICD codes used to identify CVD incidences.

| Event | Codes | |
| --- | --- | --- |
| **CVD related mortality** | Primary Cause of Death* | |
|  | 23, 25, 26, 27, 28, 29, 30, 31, 32, 35, 36 | |
| **CVD related emergency department visits and hospitalization** | ICD-9 | ICD-10 |
| *Hypertensive disease* | *402, 404* | *I11, I13* |
| *Ischemic heart disease* | *410, 411, 412, 413, 414* | *I20, I21, I22, I23, I24, I25.* |
| *Heart failure, cardiac arrest, and related* | *398, 420, 421, 422, 423, 424, 425, 426, 427, 428, 429* | *I09, I30, I31, I32, I33, I34, I35, I36, I37, I38, I39, I40, I41, I42, I43, I44, I45, I46, I47, I48, I49, I50, I51.* |
| *Cerebrovascular* | *430, 431, 432, 433, 434, 435, 436, 437, 438* | *I60, I61, I62, I63, I64, I65, I66, I67, I68, I69, G45, G46.* |

* The primary cause of death codes are based on the CMS 2746 form list of causes.

**Supplemental Table 2**. United States, 2011-2016 ZIP code-level daily ambient PM_2.5_ concentration and meteorological data for all person-days included.

1. Mortality analysis (n = 193,121,928 person-days)

|  | Mean (STD) | Range | Median | Q1 | Q3 |
| --- | --- | --- | --- | --- | --- |
| PM_2.5_ (µg/m^3^) | 8.5 (4.8) | 0,175.3 | 7.6 | 5.4 | 10.6 |
| Temperature (°C) | 15.6 (9.7) | -34.7, 41.5 | 17.1 | 9.0 | 23.3 |
| Relative Humidity (%) | 65.7 (15.7) | 0, 121.0 | 67.7 | 56.7 | 76.8 |

1. CVD incidence analysis (n = 94,385,773 person-days)

|  | Mean (STD) | Range | Median | Q1 | Q3 |
| --- | --- | --- | --- | --- | --- |
| PM_2.5_ (µg/m^3^) | 8.6 (4.9) | 0, 175.3 | 7.7 | 5.4 | 10.7 |
| Temperature (°C) | 15.6 (9.7) | -31.5, 41.5 | 17.1 | 9.1 | 23.3 |
| Relative Humidity (%) | 65.4 (15.9) | 0, 121.0 | 67.4 | 56.3 | 76.6 |

**Supplemental Table 3**. Hazards ratios (95% confidence interval) estimates per 10 µg/m^3^ increase for the association between short-term PM_2.5_ exposures and all-cause mortality, CVD-specific mortality, and incident CVD events among the overall study population.

| Outcome | Model | Lag | HR (95%CI) |
| --- | --- | --- | --- |
| ***All-cause Mortality*** | Same Day Only | Lag 0 | 1.03 (1.02, 1.05) |
|  | Lagged | Lag 0 | 1.01 (0.99, 1.03) |
|  |  | Lag 1 | 1.03 (1.01, 1.06) |
|  |  | Lag 2 | 1.00 (0.97, 1.02) |
|  |  | Lag 3 | 1.00 (0.98, 1.02) |
|  | Average Lagged | Lag 0-1 | 1.04 (1.03, 1.06) |
|  | Average Lagged | Lag 0-2 | 1.05 (1.03, 1.06) |
|  | Average Lagged | Lag 0-3 | 1.05 (1.03, 1.06) |
| ***CVD-specific Mortality*** | Same Day Only | Lag 0 | 1.04 (1.02, 1.07) |
|  | Lagged | Lag 0 | 1.02 (0.99, 1.05) |
|  |  | Lag 1 | 1.03 (0.99, 1.07) |
|  |  | Lag 2 | 0.99 (0.96, 1.03) |
|  |  | Lag 3 | 1.01 (0.98, 1.04) |
|  | Average Lagged | Lag 0-1 | 1.05 (1.03, 1.08) |
|  | Average Lagged | Lag 0-2 | 1.06 (1.03, 1.08) |
|  | Average Lagged | Lag 0-3 | 1.06 (1.03, 1.08) |
| ***Incidence CVD*** | Same Day Only | Lag 0 | 1.03 (1.02, 1.04) |
|  | Lagged | Lag 0 | 1.02 (1.01, 1.03) |
|  |  | Lag 1 | 1.01 (1.00, 1.03) |
|  |  | Lag 2 | 1.00 (0.98, 1.01) |
|  |  | Lag 3 | 1.00 (0.99, 1.01) |
|  | Average Lagged | Lag 0-1 | 1.03 (1.03, 1.04) |
|  | Average Lagged | Lag 0-2 | 1.03 (1.02, 1.05) |
|  | Average Lagged | Lag 0-3 | 1.03 (1.02, 1.04) |

**Supplemental Table 4.** LRT Test For Homogeneity Results

| **Outcome** | **Potential Modifier** | **LRT Test p-value** |
| --- | --- | --- |
| All-Cause Mortality | *Age at Dialysis Initiation* | **<0.01** |
|  | *Sex* | 0.74 |
|  | *Race* | **<0.01** |
|  | *Baseline DM* | **<0.01** |
|  | *Baseline CVD* | 0.53 |
|  | *Baseline COPD* | 0.58 |
| CVD-Specific Mortality | *Age at Dialysis Initiation* | **<0.01** |
|  | *Sex* | 0.81 |
|  | *Race* | **<0.01** |
|  | *Baseline DM* | **<0.01** |
|  | *Baseline CVD* | 0.29 |
|  | *Baseline COPD* | 0.50 |
| Incident CVD Events | *Age at Dialysis Initiation* | 0.37 |
|  | *Sex* | 0.18 |
|  | *Race* | 0.50 |
|  | *Baseline DM* | 1.00 |
|  | *Baseline CVD* | 1.00 |
|  | *Baseline COPD* | 0.14 |

**Supplemental Table 5**. Study cohort age at dialysis initiation distribution (%) by potential modifiers.

|  | ***Age Categories*** | | | |
| --- | --- | --- | --- | --- |
|  | ***18-44*** | ***45-64*** | ***65-74*** | ***75-older*** |
| **Overall Study Population** | 10.9 | 37.9 | 26.0 | 25.2 |
| **Baseline CVD** |  |  |  |  |
| *Yes* | 5.2 | 34.0 | 29.3 | 31.4 |
| *No* | 17.1 | 42.3 | 22.4 | 18.2 |
| **Baseline DM** |  |  |  |  |
| *Yes* | 8.6 | 41.8 | 28.3 | 21.4 |
| *No* | 14.1 | 32.4 | 22.8 | 30.6 |
| **Baseline COPD** |  |  |  |  |
| *Yes* | 2.2 | 31.0 | 34.3 | 32.6 |
| *No* | 11.8 | 38.7 | 25.1 | 24.3 |
| **Race** |  |  |  |  |
| *Black* | 17.1 | 45.1 | 22.1 | 15.8 |
| *White* | 8.0 | 34.5 | 28.0 | 29.5 |
| *Other* | 12.2 | 39.7 | 24.3 | 23.9 |
| **Gender** |  |  |  |  |
| *Male* | 11.5 | 39.6 | 24.8 | 24.1 |
| *Female* | 10.1 | 35.6 | 27.7 | 26.7 |
